# 

**Published:** 1859-10-01

**Authors:** 


### INDEX TO VOL. XII.

AÏSSAOUA, The, xxxix.
Arbères, Bluet d', 2
Arlidge, Dr., on Asylums of Italy, France, &c., 548
Asylums of Italy, France, Lyons, &c., by Dr. Arlidge, 548
Atkinson James, trial of, for murder, 146

BELFAST revival and hysteria, 612
Boismont, B. de, on insanity of children, 130
———— on hallucinations, Hulme's translation, xliv.
Brain, on inflammatory affections of, 517
Brougham, Lord, on popular literature, iii.

CALMEIL, on inflammatory affections of the brain, 517
Commotion, popular *v.* reform, xvi.
Convulsion, on, 89
Criminals, obscure mental disorders of, 65.
Criminals, professional, lxii.
Crime, æsthetics of, viii.
——— decrease of graver kinds, lxii.
——— as a profession, lxii.
Crofton, Capt., on intermediate system of prison discipline, xii.

DANTE, a psychological study, 413
Davey, Dr., on the ganglionic system, 141
Delepierre, Octave, on literary fools, 2—169
Devergie, Dr. A., on transitory homicidal mania, 533.
Dreams, singular result of, xxv.

EDUCATION *v.* Crime, vi.
———— early mental, principles of, 357.
———— of imbecile children, xxi.
———— in Ireland, vii.
———— Schools, artificial production of stupidity in, 186

England, state of lunacy in, 603
Epilepsy, Dr. Radcliffe on, 90
Ewings, Miss Phœbe, case of, 574

FLETCHER *v.* Fletcher, 567
Fools, Literary, 2—169
France, judicial psychology in, 223

GANGLIONIC nervous system, Dr. Davey, on, 141
General paralysis, on, 253

HOUDIN'S, Robert, memoirs, xxx.
Hallucinations, B. de Boismont, on, Hulme's translation, xliv.
——— from morphia, xlv.
Hamilton, Sir Wm., lectures on metaphysics, 314
Hoppus, Professor, on the psychology of Kant, 39, 403
Humboldt, reputed presentiment of his death, xlvi.
Hysteria and the Belfast revival, 612

INSANITY of children, by B. de Boismont, 130
———— Criminals, obscure mental disorders of, 65
———— in England, 1858,
———— heritage of, xix.
———— in Ireland, 106
———— pauper, 337
———— transitory homicidal, 533
———— puerperal, 9
———— in Scotland, 421
———— Statistics of, in France, 299
———————— in Ireland, 307
Ireland, state of lunacy in, 106
Irish revival, xlvii.

JAMAICA lunatic asylum, lxiv.
Jebb, Col., on intermediate system of prison discipline, xiv.
Johnson; Terrington *v.* Terrington and Johnson, 441
Judicial psychology in France, 223
Justice, statistics of, 75

KANT, psychology of, by Professor Hoppus, 39, 483
Kempelen's automaton chess-player, xxxviii.

LANE; Robinson v. Robinson and Lane, xxvi.
Law and Lunacy, 570
Legislation in Lunacy, proposed, 310—378
——— Scotland, 429
Literature, popular, Lord Brougham on, iii.
London, moral therapeutics of, 277
Lunacy, proposed legislation in, 310—378
Lyons, Asylums of, by Dr. Arlidge, 548

MANIA, homicidal, or murder? ix.—xlix.
——— transitory homicidal: when does reason end or mania begin? 533
——— homicidal, xlix.—lviii.
Maryborough Asylum, assault on physician, 311
MEDICO-LEGAL TRIALS:
Atkinson, James, for murder, 146
Ewings, Miss Phœbe, case of, 574
Fletcher v. Fletcher, 567
Robinson v. Robinson and Lane, xxvi.
Ruck v. Stilwell and Another, 571--615
Terrington v. Terrington and Johnson, 441
Metaphysics, Sir William Hamilton's lectures on, 314
Mind, human, Dr. Noble, on, 134
Morejon, Dr., on Don Quixote, 116
Morphia, hallucinations from, xlv.
Murder, a psychological study, xvi.
——— etiology of, lv.
Murderers, confessions of, lv.

NERVOUS system, ganglionic, Dr. Davey on, 141
Noble, Dr., on the human mind, 134

PARALYSIS, General, on, 253
Parliamentary committee on lunatics, 614
Pauper lunacy, 337
Presentiment, xlvi.
Prisons, intermediate system of discipline in, xi.
Puerperal insanity, 9

QUIXOTE, Don, a psychological study, 116

RADCLIFFE, Dr., on epilepsy and convulsive affections, 90
Redgrave, Mr. Saml., on judicial statistics, 77
Reform v. popular commotion, xvi.
Report (thirteenth) of the Commissioners of Lunacy, 614
Revival in Ireland, xlvii.
Robinson v. Robinson and Lane, xxvi.
Ruck, case of, 446
——— v. Stilwell and another, 571—615

SCOTLAND, law of lunacy and condition of insane in, 429
Second Sight, xxxv.
Sleight-of-hand, xxxiii.
Statistics of justice, 75
——— of suicide, 209
Stupidity, artificial production of, in schools, 186
Suicide, æsthetics of, 582
——— method and statistics of, 209
——— distribution of, in England, 470
——— Episode of, lix.
Sussex lunatic asylum, 463

TERRINGTON v. Terrington and Johnson, 441

VAUCANSON'S duck, xxxvii.

WITCHCRAFT in Sweden, i.

END OF VOL. XII.

